# The impact of psychological user characteristics on adherence to an mHealth intervention among women in their postpartum period

**DOI:** 10.1016/j.invent.2026.100968

**Published:** 2026-06-22

**Authors:** Natalie Schoemann, Julia C. Hügel, Lea Vogel, Tom Deliens, Johanna Löchner, Ulrike Lux, Ansgar Opitz, Caroline Seiferth, Jörg Wolstein, Carmen Henning

**Affiliations:** a Department of Psychopathology, University of Bamberg, Bamberg, Germany; b Department of Psychology, LMU Munich, Munich, Germany; c German Center for Mental Health (DZPG), partner site, Munich, Germany; d Department of Movement and Sport Sciences, Faculty of Physical Education and Physiotherapy, Vrije Universiteit Brussel, Brussels, Belgium; e Institute for General Practice and Family Medicine, Ludwig-Maximilians-University, University Hospital, Munich, Germany; f Department of Families and Family Policies, National Center for Early Prevention, German Youth Institute, Munich, Germany; g Freie Universität Berlin, Department of Clinical Psychology and Psychotherapy, Berlin, Germany; h German Centre for Mental Health (DZPG), partner site, Berlin-Potsdam, Germany

**Keywords:** Adherence, mHealth, Postpartum, Mental health

## Abstract

Until now, the impact of psychological factors on adherence to mHealth applications, especially among postpartum women, remains poorly understood, even though the efficacy depends on the regular use of the applications. The I-PREGNO application aims to promote maternal healthy weight management and improve mental health during the perinatal period by addressing core psychological skills like stress management and emotion regulation. This study examined whether depressive symptoms, parenting stress, emotion regulation difficulties, self-efficacy, and partnership status predicted adherence across four dimensions (length, breadth, depth, interaction) in a pooled sample of *N* = 154 postpartum women from two randomized controlled trials, using hierarchical regression analyses and latent profile analysis.

No psychological predictor survived correction for multiple testing, and the overall pattern of results does not support the conclusion that baseline psychological characteristics reliably determine adherence in this population. Before correction, isolated associations emerged: higher depressive symptoms with greater interaction (number of character input, *IRR* = 1.10, 95% CI [1.00, 1.21]), higher self-efficacy with lower depth (using the app at least three times per week, *IRR* = 0.64, 95% CI [0.40, 1.02]), and partnership status with longer usage (time spent in the app, *B* = 1.90, 95% CI [0.32, 3.47]) but these should be treated as preliminary signals, particularly given the limited statistical power. Latent profile analysis identified three adherence patterns: low (24.0%), moderate (60.4%), and high adherence (15.6%). Psychological factors did not significantly differentiate between adherence patterns. The findings do not provide robust evidence that baseline psychological characteristics predict adherence to mHealth interventions in postpartum women. Future research should consider more proximal determinants of adherence, including social, structural, and intervention-related factors.

## Highlights

• Multidimensional mHealth adherence assessed (length, depth, breadth, interaction)
• Psychological baseline characteristics did not predict mHealth adherence
• No robust links between psychological variables and adherence dimensions or classes
• Three adherence patterns emerged (low, moderate, high) in latent profile analysis
• Adherence likely depends on factors beyond psychological baseline characteristics

## 1 Introduction

In recent years, the number of available mobile Health (mHealth) applications (apps) increased significantly, offering cost-efficient and easy access to various health services and health-related information (Goldberg et al., 2022; Seiferth et al., 2023). These advantages make mHealth apps particularly valuable for postpartum women, as they help address common challenges like time constraints during this phase of life (Attard et al., 2022). Considering specific problems and issues emerging during the postpartum period, such as a substantial rate of depression or the risk of weight retention (Saxbe et al., 2018), the provision of needs-based support services is essential. Evidence suggests that digital health interventions can be effective in reducing (Goldberg et al., 2022) and preventing depressive symptoms (Watkins et al., 2024) and promoting weight loss (Herring et al., 2014) in the postpartum period. Combined with positive evaluations from app users (Lim et al., 2019), this suggests that mHealth interventions could serve as an effective means of providing support for postpartum women.

Despite the benefits of mHealth apps, high attrition and low adherence – defined as the extent to which participants engage with an intervention as intended (Kernebeck et al., 2021) – remain widely recognized challenges (Kelders et al., 2013). Difficulties concerning adherence are problematic among postpartum women as well (Gilmore et al., 2017). Adherence to technology-based lifestyle interventions tends to decline over time in this group (Christiansen et al., 2019), with most users classified as low or moderate adherers (Gilmore et al., 2017). Insufficient engagement can result in reduced effectiveness of the interventions (Vandelanotte et al., 2016; Vitolins et al., 2000), thus underscoring the importance of understanding the factors influencing adherence to improving the impact of mHealth interventions (Lim et al., 2019).

Adherence measures vary across studies, making comparisons difficult (Sieverink et al., 2017). To address this issue, Yang et al. (2022) propose four dimensions of adherence: length (frequency or duration of app use), breadth (engagement with different app features), depth (goal achievement), and interaction (active or passive participation). While similar to adherence, engagement encompasses both the user's experience and their level of app usage (Perski et al., 2017). Given their overlap, predictors of engagement thus may also influence adherence (Kelders et al., 2013).

Recent research has explored various factors influencing adherence (Yang et al., 2022) and related constructs like engagement (Perski et al., 2017). Perski et al. (2017) suggest that engagement with digital interventions is shaped by demographic (e.g. age, education), physical (e.g. physique), and psychological factors (e.g., mental health, self-efficacy). Similarly, Yang et al. (2022) identify three categories of adherence predictors for mHealth apps fostering physical activity: user characteristics (e.g. age, weight, education), technology-related factors (e.g., reminders; Ammenwerth et al. (2023)), and contextual factors (e.g. weekdays vs. weekends; Jakob et al. (2022)). The present paper focuses on user characteristics, as they are potential determinants of adherence, influencing how individuals engage with and sustain their participation in mHealth, thereby shaping its overall effectiveness.

Prior research on user characteristics has primarily focused on sociodemographic variables such as age, gender, education, and income (Beatty and Binnion, 2016; Jakob et al., 2022; Reinwand et al., 2015; Yang et al., 2022). However, emerging evidence suggests that psychological factors may also play a crucial role in determining adherence to mobile health (mHealth) applications (Elavsky et al., 2017). To derive psychological predictors of adherence to mHealth interventions, several behavior change models can be applied. Yeager and Benight (2018) adapted the Health Action Process Approach (Schwarzer, 2008) to predict engagement with digital health interventions, emphasizing that both barriers and resources influence whether intended health behaviors are implemented. Similarly, the Capability–Opportunity–Motivation–Behavior (COM—B) model (West and Michie, 2020) and the adapted Behavioral Model of Health Service Use (Bina, 2020) highlight that motivation, available resources, and contextual opportunities determine whether health-related behaviors are enacted. Transferred to adherence to mHealth applications, these frameworks suggest that engagement may be shaped by several underlying mechanisms, including motivation and goal pursuit (e.g. the ability to initiate and sustain intended behaviors), cognitive load and self-regulatory capacity (e.g. the ability to manage emotions), and resource availability (e.g. social support and time).

Building on these frameworks, the current study conceptualized several individual characteristics that may influence these mechanisms. Specifically, depressive symptoms, parenting stress, emotion regulation difficulties, self-efficacy, and partnership status were considered individual-level barriers or resources that may shape engagement with the app across the four adherence dimensions: length, breadth, depth, and interaction. These predictors were chosen as indicators of the underlying mechanisms highlighted by the models above, namely, motivational (e.g., depressive symptoms, self-efficacy), self-regulatory (e.g., emotion regulation difficulties), and resource availability (e.g., parenting stress, partnership support) that may facilitate or hinder adherence to digital health interventions.

First, higher levels of depressive symptoms such as low mood, inactivity, and cognitive deficits (Dorsch and Rohde, 2016) may function as barriers. Both are associated with reduced motivational capacity and impaired goal pursuit (Dickson et al., 2016), potentially making it more difficult to initiate and sustain intended app use over time (length, depth), to complete a broader range of sessions or modules (breadth), and to engage actively with exercises (interaction) (Makama et al., 2021). However, findings on the relationship between depressive symptoms and mHealth adherence (different operationalizations) are mixed, with some studies showing negative (Arean et al., 2016; Christensen et al., 2009; Jakob et al., 2022), positive (Mikolasek et al., 2018), or no significant associations (Hung et al., 2016; Trompetter et al., 2015).

Second, parenting stress may impose situational and emotional constraints that compete with app use. Parenting demands reduce available time and cognitive resources, and more time constraints have been linked to lower adherence in mHealth trials (Jakob et al., 2022). For postpartum women, high stress may particularly compromise the ability to engage at all (length), regularly (depth) and broadly with app content (breadth), and to complete more demanding exercises (interaction).

Emotion regulation difficulties represent limitations in self-regulatory capacity. Adaptive emotion regulation supports goal-directed behavior, while maladaptive emotion regulation (e.g. avoidance, rumination) may disrupt the process, potentially reducing adherence (Thompson, 2019). In digital interventions, poor emotional regulation capacity is therefore expected to impede sustained and effortful app use (length, depth), exploration of available content (breadth), and willingness to engage in emotionally demanding exercises (interaction).

Individuals with higher self-efficacy are expected to demonstrate greater engagement, persistence (Bandura, 1982), motivation (Demir, 2020) and problem-solving skills (Hoffman and Schraw, 2009). In the context of digital interventions, higher self-efficacy may therefore facilitate sustained engagement with the app (length), exploration of a wider range of available content (breadth), more thorough completion of modules (depth), and greater willingness to engage in interactive exercises (interaction).

Similarly, a supportive partnership can serve as a motivational resource (Liu et al., 2022) and support postpartum women with time consuming child demands (Makama et al., 2021; Mitchell et al., 2018). As social support is perceived as a facilitator of mHealth use (Lim et al., 2019), it may foster sustained engagement (length), more consistent use (depth), broader exploration of app content (breadth), and greater willingness to complete demanding exercises (interaction). Previous studies indicate that both partnership status (Reinwand et al., 2015) and self-efficacy (Jakob et al., 2022) are positively associated with adherence, though evidence regarding the latter remains mixed (Beatty and Binnion, 2016).

Research on psychological factors influencing mHealth adherence in postpartum women remains limited with only few studies conducted in other populations, such as individuals with depressive symptoms (Arean et al., 2016; Christensen et al., 2009; Hung et al., 2016), cancer patients (Mikolasek et al., 2018), and those with chronic pain (Trompetter et al., 2015), that have explored these associations.

To address this gap, our current study examines psychological predictors of adherence to the I-PREGNO mHealth app. The I-PREGNO app aims at preventing unhealthy weight management and promoting mental health during the perinatal period, offering the possibility to enhance the understanding of adherence patterns in postpartum women. Particularly developed to also reach psychosocially burdened parents (e.g., families facing mental health challenges; (Vogel et al., 2024), the app provides tailored, time-sensitive content for pregnancy and the postpartum period. As part of the I-PREGNO project, the healthcare professional study offered psychosocially burdened families access to the app alongside regular counseling from healthcare professionals (Vogel et al., 2023). Additionally, a self-guided study arm allowed broader testing of the app, including families with and without psychosocial burden (Henning et al., 2023).

Based on the above-mentioned literature, it is hypothesized that specific psychological factors predict higher adherence to the I-PREGNO mHealth app among postpartum women. Specifically, it is expected that

• lower depressive symptoms,
• lower parenting stress,
• fewer difficulties in emotion regulation,
• higher self-efficacy, and
• having a partner

will be associated with greater adherence, as measured by length, breadth, depth, and interaction with the app, after controlling for potential confounders (maternal age, education, Body Mass Index (BMI), child age and type of intervention). These analyses were theoretically informed but were ultimately exploratory in nature.

In addition, an exploratory analysis will investigate whether distinct adherence classes (length, breadth, depth, interaction) can be identified among participants and psychological predictors of adherence class membership will be investigated exploratorily to determine whether they differentiate between the identified adherence classes.

## 2 Method

This secondary analysis pooled data from two distinct randomized controlled trials (RCT) conducted within the I-PREGNO project (https://osf.io/s28n7/): a cluster randomized controlled trial (cRCT) with blended counseling (Vogel et al., 2023) and a self-guided RCT (Henning et al., 2023). The project received ethical approval from the ethical committee of the University in Bamberg (nr. 2022–02/09). The analyses and hypotheses of this paper were registered in the Open Science Framework (https://osf.io/7nu3r). Although the preregistration was uploaded after the analyses had been conducted, the analysis plan was fully specified a priori. However, given the large number of hypotheses (k = 20) and the post hoc timing of preregistration, the study is best characterized as primarily exploratory in nature. Data collection began in October 2022 for the cRCT and in March 2023 for the self-guided RCT, concluding in February 2024 for both.

### 2.1 Procedure

In the cRCT, healthcare professionals (e.g., family midwives, family nurses) within the national early childhood intervention program representing the clusters of the cRCT, were recruited via emails and online advertising. Healthcare professionals were randomly assigned to one of two initial groups: blended counseling (I-PREGNO app and counseling within the regular home-visiting program), or treatment as usual (home-visiting program only). Afterwards healthcare professionals screened families for eligibility, targeting those with psychosocial burdens like financial difficulties, single parenthood, or parental mental illness (for a more detailed list, see Vogel et al. (2023)).

The self-guided trial differed in that participants were not required to have psychosocial burdens and that the recruitment occurred via social media, press releases, leaflets, and posters in child-focused locations like kindergartens and pediatric practices. Interested individuals completed an online questionnaire to determine eligibility (see below) and were then randomly assigned to the I-PREGNO intervention or wait-list control group (Henning et al., 2023).

Eligible participants completed online questionnaires at three time points: baseline (t0), approximately 12 weeks later (t1), and six months after t0 (t2). In this study, only data from the intervention groups from both studies at t0 and t1 were used. Between t0 and t1, intervention groups received their assigned interventions: app access with counseling in the blended counseling study or without in the self-guided trial. After t2, the participants could opt to reactivate app access. Participants completing t0 and t1 were compensated with €20. Healthcare professionals received €50 for each family successfully recruited. Detailed study procedures are described in the respective protocols (Henning et al., 2023; Vogel et al., 2023).

### 2.2 Participants

Both studies shared the same inclusion and exclusion criteria, except for the psychosocial burden requirement in the cRCT (Henning et al., 2023; Vogel et al., 2023). Mothers needed to have a child aged 0–12 months, own a smartphone, be at least 16 years old, and speak German. Exclusion criteria included severe mental health issues or other factors preventing successful participation, as well as chronic illnesses like diabetes that could influence behavior related to energy balance.

### 2.3 Intervention

During the intervention period of approximately 12 weeks (blended counseling study: 84 days; self-guided study: 85 days), participants in both studies had the opportunity to use the mHealth app I-PREGNO (Henning et al., 2023; Vogel et al., 2023). Although a usage frequency of at least three times per week was recommended, interactions with the app were self-directed. Additionally, participants in the blended counseling study received some short counseling sessions regarding app usage within the regular home-visiting program (treatment as usual) from their healthcare professionals. For details on the blended counseling setting see Vogel et al. (2023).

The I-PREGNO app is based on cognitive behavioral therapy (CBT) and behavior change techniques (Vogel et al., 2023). Tailored for pregnancy and postpartum needs, the app features modules addressing psychological skills such as self-esteem, stress management, emotion regulation, self-efficacy, social skills, and mindfulness, as well as modules on nutrition, physical activity, and a tutorial.

The postpartum version features 12 modules, each comprising 2–19 sessions (totaling 100 sessions), delivered through texts, podcasts, and interactive features like input fields and mini-games. Users can also track progress via self-monitoring tools and save preferred topics. Further details are available in Appendix A.

### 2.4 Measures

For the current analyses, baseline (t0) and intervention-period usage data were utilized as adherence predictors. Sociodemographic variables, including maternal and child age, education level, and self-reported weight and height, were collected at t0. Usage data were recorded from October 2022 to September 2023 in the blended counseling study and from March to October 2023 in the self-guided trial. Both studies relied on self-report questionnaires, with the blended counseling study also including psychosocial burden reported by healthcare professionals. Usage data were securely stored by the external software provider groupXS Solutions GmbH.

### 2.5 Sociodemographic variables

Education levels were categorized into three groups based on International Standard Classification of Education (ISCED) guidelines (International Standard Classification of Education (ISCED*)*, n.d.): low (no school-leaving qualification or about nine years of schooling), medium (about 10–13 years of schooling), and high (university degree), using the high education group as the reference category in regression analyses. BMI was calculated as weight (kg) divided by the square of height (m^2^).

### 2.6 Psychological variables

Postpartum depressive symptoms were assessed using the 10-item Edinburgh Postnatal Depression Scale (EPDS) (Bergant et al., 2008; Cox et al., 1987). Items were rated on a 4-point Likert scale (0–3), with total scores ranging from 0 (no symptoms) to 30 (severe symptoms). The scale demonstrated good internal consistency (Cronbach's α = 0.86).

Parenting stress was assessed using the German Parenting Stress Index-Short Form (PSI-SF; Abidin et al., 2006; Tröster, 2011), which measures social isolation, partner relationship, health, parental attachment, role restriction, parenting competence, and depression. Each of the 28 items was rated on a 5-point Likert scale (1 = “strongly disagree” to 5 = “strongly agree”), with higher sum scores indicating greater parenting stress. Partner relationship items were excluded to ensure comparability between mothers with and without a partnership. Internal consistency was excellent (Cronbach's α = 0.92).

The short form of the Difficulties in Emotion Regulation Scale (DERS-SF; Gratz and Roemer, 2004) assesses emotion regulation difficulties using 18 items rated on a 5-point Likert scale (1 = “almost never” to 5 = “almost always”). It includes six subscales: awareness, clarity, nonacceptance, goals, impulse, and strategies. Higher sum scores indicate greater difficulties in emotion regulation. Internal consistency was excellent (Cronbach's α = 0.90).

General self-efficacy was assessed using the German version of the General Self-Efficacy Scale (GSE; Schwarzer et al., 1995; Schwarzer and Jerusalem, 2010). The scale consists of ten items rated on a 4-point Likert scale (1 = “not at all true”, 2 = “hardly true”, 3 = “moderately true”, 4 = “exactly true”). Mean scores were calculated, with higher scores indicating greater self-efficacy. Internal consistency was good (Cronbach's α = 0.89).

Partnership status was assessed with the item “Do you currently have a partner?” and coded dichotomously (yes = 1, no = 0).

### 2.7 Adherence

Adherence was assessed using the framework by Yang et al. (2022), encompassing four dimensions: length, breadth, depth, and interaction.

Length was defined as the total time spent using the I-PREGNO app (in minutes). Time was reconstructed from timestamped event logs. Navigation events containing a module name were identified, and transitions between modules (entry, exit, or switch) were marked as “edges.” Continuous activity blocks were calculated as the time difference between consecutive edge events within the same module and summed per participant. The app automatically generated additional system events: *pause* occurred when the app was minimized, sent to the background, or when the device screen was turned off while the app was open. *Resume* occurred when the app was brought back to the foreground, reopened, or when the device screen was unlocked. Pause and resume events were not modeled as separate time segments. Instead, they typically resulted in a module-to-empty transition, which created an edge and thus ended the active module block. When the user returned to the app and triggered a new navigation event, a new activity block began. In this way, background time (i.e. time when the app was not actively visible) was largely excluded from duration. To reduce bias from logging artifacts or unrecorded pauses, activity blocks exceeding 60 min were excluded. Time spent therefore represents a reconstructed approximation of active in-app engagement rather than exact screen time.

Breadth was operationalized by the number of completed sessions (100 sessions available). App content was hierarchically structured into modules, slots, sessions, and sections. A session was defined as the complete traversal of all sections within a slot of a module. Session completion was operationalized via an event-log variable, which was triggered only when users actively clicked the final button on the last section and returned to the slot overview. Completion was therefore based exclusively on this explicit action and not inferred from timestamps or passive page traversal. Consequently, the number of completed sessions may be conservatively underestimated if users completed the final section but exited without tapping the final button.

Depth was operationalized as the number of intervention weeks in which participants met the predefined usage recommendation, defined as three separate login events recorded within a given intervention week. Login events were automatically generated by the app each time a participant actively opened and accessed the application. The depth score therefore reflects the consistency of engagement across the intervention period rather than overall frequency of use.

Interaction was evaluated based on the number of characters entered in open text boxes (e.g. answers to questions and diary entries). This metric captures active content-related engagement beyond passive navigation or module completion.

All adherence outcomes were derived from meta-data log files, which were downloaded to secure servers after recording usage data within the app (Henning et al., 2023).

### 2.8 Statistical analyses

Analyses were performed using R (Version 4.3.3) and RStudio (Version 2024.04.0), with applied packages listed in Appendix B. Frequencies, means, and standard deviations were calculated to describe the sample characteristics and adherence patterns of each trial as well as the pooled sample. Welch's *t*-tests for independent samples were used to assess differences between the blended-counseling and self-guided studies for metric variables, accounting for unequal variances. Normal distribution was assumed due to the large sample size (*n* > 30) (Sainani, 2012). *χ*^2^ tests were conducted for dichotomous variables, and Fisher-Yates tests were performed when expected cell frequencies were too low, following recommendations by Gries (2023).

Two-step hierarchical regression analyses were used to identify predictors of adherence to the I-PREGNO app, with separate models for each adherence measure (four models total). In linear and negative binomial regression models, control variables were entered first (maternal age, education, BMI, child age, type of intervention; Model 1), followed by psychological factors (depressive symptoms, parenting stress, difficulties in emotion regulation, self-efficacy and having a partner) in a second step (Model 2). *P*-values < .05 were initially considered statistically significant. To account for the increased risk of false positives due to multiple testing, *p*-values were adjusted within each family of predictors using the Benjamini–Hochberg false discovery rate (FDR) procedure. This adjustment was not preregistered but was implemented to enhance the robustness of statistical inferences.

Regarding the adherence dimensions, descriptive analyses indicated positive skewness for length (skew = 2.64, kurtosis = 7.19). Residuals from an initial linear model indicated heteroscedasticity (Breusch-Pagan test: length step 1 BP *χ*^2^(6) = 13.60, *p* = .034; length step 2 BP *χ*^2^(11) = 20.80, *p* = .036). To address this, a logarithmic transformation (log(x + 1)) was applied (Bartlett, 1947), after which residual variance was approximately constant (Breusch-Pagan test: Length *χ*^2^(6) = 3.88, *p* = .693; *χ*^2^(11) = 8.86, *p* = .635). Robust linear regression was subsequently used to account for extreme values, and visual inspection of residuals indicated near-normal distribution.

For breadth, depth, and interaction, hierarchical negative binomial regression models were used, as recommended by Coxe et al. (2009) and O'Hara and Kotze (2010) as these dimensions showed overdispersion without substantial zero-inflation: Descriptive analyses indicated positive skewness for breadth (skew = 1.39, kurtosis = 0.69), depth (skew = 1.95, kurtosis = 3.41), and interaction (skew = 3.55, kurtosis = 15.13). The outcomes showed substantial overdispersion, as indicated by variances markedly exceeding their means (breadth: *M* = 21.98, Var = 725.22; depth: *M* = 2.07, *Var* = 7.40; interaction: *M* = 1844.21, *Var* = 12,330,857.86). Zero-inflation diagnostics were conducted by comparing observed versus model-predicted zeros. Ratios of observed to predicted zeros ranged from 0.65 to 1.11 across outcomes, with all *p*-values > .05, indicating that the models adequately captured the frequency of zero counts. Therefore, hierarchical negative binomial regression models were estimated instead of Poisson models, as they account for overdispersion and provide robustness against extreme entries. As a sensitivity analysis, the interaction outcome was winsorized at the 95th percentile to reduce the influence of extreme values due to its large variance. The fully adjusted negative binomial regression model was then re-estimated using the winsorized outcome.

Maternal age, education, BMI, child age, and intervention type (blended counseling or self-guided trial to account for structural differences such as e.g. human support) were included as control variables due to their associations with adherence, mHealth use, and postpartum depressive symptoms, as well as differences in study design (Dol et al., 2021; Elavsky et al., 2017; Henning et al., 2023; Jakob et al., 2022; Vogel et al., 2023). Exploratory subgroup analyses by intervention type were attempted; however, these models were not stable and did not converge reliably due to small sample sizes and high overdispersion. Therefore, to assess subgroup differences, descriptive sensitivity analyses comparing pooled adjusted models, pooled unadjusted models, and trial-specific estimates were conducted. Further sensitivity analyses were conducted to evaluate the ability of the available sample size (*N* = 154) to detect small and medium effects. Analyses were performed using G*Power 3.1.9.7 for multiple linear regression (Faul et al., 2009) and simulation-based methods in R (Version 4.3.3) for negative binomial regression.

To identify distinct patterns of adherence, a Latent Profile Analysis (LPA) was conducted using the *depmixS4* package (Visser and Speekenbrink, 2010) and following the approach of Fortunato et al. (2023). Four indicators were included: length (total duration of app use in minutes), breadth (number of completed sessions), depth (number of weeks used as recommended), and interaction (total number of characters entered). Prior to analysis, all indicators were log-transformed using a log(*x* + 1) transformation to address positive skew and subsequently z-standardized. Models with one to five latent profiles were estimated using Gaussian response distributions, with 20 random starts per model to avoid local optima. Model selection was based on AIC, BIC and minimum class size. Classification accuracy was evaluated using average posterior probabilities per class. As a sensitivity analysis, the LPA was rerun using mixed response distributions: a Gaussian distribution for log-transformed length, and Poisson distributions for breadth, depth, and interaction. The latter three indicators were rescaled to a 1–13 range (similar to the depth range) for comparability.

Finally, a series of multinomial logistic regression models were conducted to examine the relationship between psychological predictors (depressive symptoms, parenting stress, difficulties in emotion regulation, self-efficacy and having a partner) and the likelihood of membership in the three latent classes. The McFadden's *R*^*2*^ was used for model evaluation.

## 3 Results

### 3.1 Descriptive statistics

Out of 395 participants (blended counseling study: *n* = 110; self-guided trial: *n* = 285), *n* = 184 assigned to the intervention groups were included (blended counseling study: *n* = 46; self-guided trial: *n* = 138) due to the need for usage data to analyze adherence. Three women in the blended counseling study were excluded because of dropping out before accessing the app (blended counseling study: *n* = 43). After excluding 24 fathers (blended counseling study: *n* = 4; self-guided trial: *n* = 20) to focus on postpartum women, the final intervention group consisted of 39 mothers (blended counseling study) and 118 mothers (self-guided trial), totaling 157 female participants. Three cases with missing covariate values were excluded, resulting in a final sample of *N* = 154 (blended counseling study: *n* = 37; self-guided trial: *n* = 117).

Maternal age, BMI, and depressive symptoms differed significantly between the two study types at baseline (see Table 1). The samples also differed significantly in terms of education and partnership: high education (*χ*^2^(1) = 43.83, *p* ≤0.001, Cramér's *V* = 0.55), medium education (*χ*^2^(1) = 9.09, *p* = .003, Cramér's *V* = 0.26), low education (*OR* = 22.43, *p* ≤0.001, 95% CI [5.66, 131.27]), and partnership (*OR* = 0.08, *p* ≤0.001, 95% CI [0.01, 0.43]).No significant differences were observed for any adherence dimension. App usage duration (length) ranged from 0 to 644.4 min, completed sessions ranged from 0 to 96 of 100 available sessions, adherence to the recommended three logins per week ranged from 0 to 12 weeks, and interaction varied widely, with text input ranging from 0 to 23,370 characters. Missing timestamps in some data inputs complicated the interaction dimension analysis, but inaccuracies are considered negligible.

**Table 1:** Sample Characteristics Overall and Arranged by Study Type and Difference Statistics for Metric Variables.

Variable	Overall (*N = 154)*	Blended counseling study (*n* = 37)	Self-guided study (*n* = 117)	*t*	*df*	*p*	Cohen's *d*
Control variables							
Age of the mother in years; *M* (SD)	32.71 (5.42)	30.14 (7.15)	33.53 (4.48)	2.72⁎⁎	45.28	0.009	0.65
Education							
High; %(*n*)	56.49 (87)	8.11 (3)	71.79 (84)				
Medium; %(*n*)	32.47 (50)	54.05 (20)	25.64 (30)				
Low; %(*n*)	11.04 (17)	37.84 (14)	2.56 (3)				
BMI in kg/m^2^; *M* (SD)	25.63 (5.91)	28.03 (8.59)	24.87 (4.55)	−2.14⁎	42.57	0.038	0.55
Child age in months; *M* (SD)	5.08 (3.20)	5.41 (3.18)	4.98 (3.22)	−0.70	61.20	0.485	0.13
Psychological variables							
Depressive symptoms; *M* (SD)	8.22 (5.56)	11.87 (6.68)	7.07 (4.62)	−4.07⁎⁎⁎	47.36	<0.001	0.93
Parenting stress; *M* (SD)	69.40 (17.41)	72.54 (22.35)	68.41 (15.51)	−1.05	47.45	0.300	0.24
Difficulties in emotion regulation; *M* (SD)	36.39 (11.89)	39.60 (13.88)	35.38 (11.06)	−1.69	51.27	0.097	0.36
Self-efficacy; *M* (SD)	2.86 (0.51)	2.82 (0.65)	2.88 (0.45)	0.51	47.71	0.614	0.11
Partnership status*;* %(*n*)	94.16 (145)	81.08 (30)	98.29 (115)				
Adherence							
Length in minutes; *M* (SD)	81.59 (123.20)	93.58 (149.67)	77.80 (114.05)	−0.59	49.91	0.558	0.13
Breadth; *M* (SD)	21.98 (26.93)	25.00 (31.61)	21.03 (25.35)	−0.70	51.47	0.489	0.15
Depth; *M* (SD)	2.07 (2.72)	2.38 (2.99)	1.97 (2.63)	−0.75	54.74	0.456	0.15
Interaction; *M* (SD)	1844.2 (3511.53)	2139.03 (4536.28)	1750.97 (3137.08)	−0.48	47.38	0.630	0.11

*Note*. *t* = t-Score of *t*-test. *df* = degrees of freedom. Depressive symptoms were measured by the EPDS, Parenting stress by the PSI-SF, difficulties in emotion regulation by the DERS-SF, Self-efficacy by the GSE. Length was measured by the total time spent using the I-PREGNO app (in minutes), breadth by the number of completed sessions, depth by the number of weeks participants met the usage recommendation (at least three logins per week), interaction was evaluated based on the number of characters entered in open text boxes.

⁎ *p* < .05.

⁎⁎ *p* < .01.

⁎⁎⁎ *p* < .001.

Table 1 presents a descriptive comparison of baseline characteristics including maternal age, education, BMI, child age in months, psychological variables, as well as adherence dimensions across both study types.

### 3.2 Regression analyses

Detailed results for the theory-informed regression analyses, including statistical values and effect sizes, are provided in Table 2. Although several unadjusted associations reached nominal significance, none remained significant after FDR correction, indicating that the following observed effects should be interpreted with caution.

**Table 2:** Results of the Regression Analyses with the Adherence Dimensions Length, Breadth, Depth, and Interaction as Dependent Variables.

Predictors	Length							Breadth							Depth							Interaction						
	Model 1			Model 2				Model 1			Model 2				Model 1			Model 2				Model 1			Model 2			
	*β*	*t*	*p*	*β*	*t*	*p*	*p(FDR)*	IRR	*z*	*p*	IRR	*z*	*p*	*p*(FDR)	IRR	*z*	*p*	IRR	*z*	*p*	*p(FDR)*	IRR	*z*	*p*	IRR	*z*	*p*	*p*(FDR)
Constant		1.23	0.220		1 .13	0.262			2.86	0.004		0.79	0.427			0.12	0.906		0.09	0.928			6.14	<0.001		4.51	<0.001	
Control Variables																												
Age	0.11	0.58	0.563	0.03	0.24	0.815		1.02	0.73	0.468	1.02		0.490		1.04	1.59	0.111	1.05	2.33	**0.020**		1.04	1.28	0.202	1.02	0.73	0.469	
Education (reference: High)																												
Medium^a^	–0.08	0.37	0.712	–0.12	–1.03	0.304		0.98	0.07	0.942	1.01	0.04	0.968		0.87	0.51	0.612	0.96	0.17	0.866		1.02	0.05	0.958	0.99	0.01	0.989	
Low^a^	–0.20	0.83	0.407	–0.10	0.71	0.477		0.78	0.52	0.605	0.82	0.39	0.695		1.00	0.00	0.998	1.18	0.36	0.723		0.33	–1.73	0.084	0.23	–2.25	**0.025**	
BMI	–0.02	0.13	0.895	–0.07	0.57	0.569		1.00	0.23	0.816	1.00	0.10	0.924		0.99	0.49	0.621	0.98	–1.01	0.311		0.96	–1.65	0.100	0.94	–2.06	**0.039**	
Age of the child	–0.01	0.06	0.950	–0.02	0.18	0.858		0.99	0.27	0.785	0.99	0.33	0.742		0.97	0.99	0.321	0.97	–1.10	0.270		0.93	–1.64	0.102	0.93	–1.68	0.094	
Type of intervention^b^	0.20	0.94	0.348	0.20	1.52	0.130		1.42	1.09	0.274	1.23	0.61	0.545		1.50	1.26	0.173	1.57	1.43	0.153		2.18	1.84	0.067	1.47	0.85	0.398	
Psychological variables																												
Depressive symptoms				0.16	0.96	0.338	0.640				1.02	0.52	0.607	0.607				0.98	0.72	0.473	0.591				1.10	2.16	0.**031**	0.155
Parenting stress				0.07	0.47	0.640	0.640				1.01	1.63	0.104	0.520				1.00	0.15	0.881	0.881				0.99	0.72	0.472	0.972
Difficulties in Emotion Regulation				–0.12	0.78	0.435	0.640				0.99	0.68	0.496	0.607				1.02	1.23	0.220	0.550				1.00	0.11	0.915	0.972
Self-efficacy				–0.06	0.57	0.570	0.640				1.14	0.54	0.587	0.607				0.64	–2.01	**0.044**	0.220				0.99	0.04	0.972	0.972
Partnership^a^				0.28	2.36	0**.020**	0.100				1.34	0.55	0.580	0.607				1.58	0.90	0.370	0.591				0.76	0.40	0.693	0.972
*R*^*2*^ / Dispersion (θ)	0.035			0.092				0.59			0.60				0.99			1.10				0.33			0.34			
Δ*R*^*2*^*/* 2 x log-likelihood				0.057				−1239.36			−1235.24				−590.23			−581.12				−2409.16			−2402.68			
Mc Fadden *R*^*2*^/Efron *R*^*2*^								0.001/0.007			0.005/0.019				0.010/0.029			0.025/0.062				0.004/0.029			0.007/0.081			

*Note*. *N* = 154; Depressive symptoms were measured by the EPDS, Parenting stress by the PSI-SF, difficulties in emotion regulation by the DERS-SF, Self-efficacy by the GSE. Length was measured by the total time spent using the I-PREGNO app (in minutes), breadth by the number of completed sessions, depth by the number of weeks participants met the usage recommendation (at least three logins per week), interaction was evaluated based on the number of characters entered in open text boxes. Control variables were entered first (maternal age, education, BMI, child age, type of intervention; Model 1), followed by psychological factors (depressive symptoms, parenting stress, difficulties in emotion regulation, self-efficacy and having a partner) in a second step (Model 2). *ß* = standardized regression coefficient; *t* = t-value of *t*-test. *p* = *p-* value*. p*(FDR) = adjusted *p-*value for multiple testing within family. IRR = incidence rate ratio. *z* = *z-*Score. Bold text indicates significant predictors before FDR correction. Unadjusted level of significance was set at *p* < .05. ∆*R*^2^ = change in *R*^*2*^.

a 0 = no, 1 = yes.

b 0 = app access without blended counseling, 1 = app access including blended counseling.

Being in a partnership was associated with the log-transformed length of app use, with partnered postpartum mothers demonstrating longer app use (*B* = 1.90, 95% CI [0.32, 3.47], *p* = .020). Depressive symptoms, parenting stress, emotion regulation difficulties, and self-efficacy were not significant predictors of length. Model 2 explained limited additional variance compared to the null model (see Table 2).

None of the predictors were significantly associated with the breadth dimension (number of completed sessions). Model 2 did not improve over the null model (*χ*^2^(11) = 5.81, *p* = .886; AIC = 1245.05 vs. 1261.24), indicating low explanatory value.

For the depth dimension (number of weeks participants met the usage recommendation), higher self-efficacy was associated with lower adherence (IRR = 0.64, 95% CI [0.40, 1.02], *p* = .044). Depressive symptoms, parenting stress, emotion regulation difficulties, and partnership status were not significant predictors. Model 2 did not significantly improve model fit over the null model (*χ*^2^(11) = 14.87, *p* = .189) and showed poorer fit based on AIC (AIC = 599.99 vs. 607.12).

Higher depressive symptoms were associated with increased interaction within the I-PREGNO app (IRR = 1.10, 95% CI [1.00, 1.21], *p* = .031)**.** No significant effects were found for parenting stress, emotion regulation, self-efficacy, or partnership status. Model 2 did not improve prediction over the null model (*χ*^2^(11) = 16.95, *p* = .109; AIC = 2428.68 vs. 2423.63). After winsorizing the interaction variable at the 95th percentile, the negative binomial models showed a comparable dispersion parameter (*θ* ≈ 0.35 in both models) but a slightly higher model fit index (AIC = 2406.7 vs. 2412.5). Effect directions were consistent with the primary analyses; however, all predictors were attenuated and none reached statistical significance (all *p* > .05), with confidence intervals crossing zero. See Appendix C for full results.

To account for the increased risk of false positives due to multiple testing, *p*-values were adjusted within each family of predictors using the Benjamini–Hochberg false discovery rate (FDR) procedure. After correction, all predictors were non-significant. The smallest FDR-adjusted *p*-values were 0.10 for partnership predicting length; 0.52 for parenting stress predicting breadth; 0.22 for self-efficacy predicting depth; and 0.16 for depressive symptoms predicting interaction. All other predictors had adjusted *p*-values ≥ .55, and none reached the conventional *α* = 0.05 threshold.

Descriptive sensitivity analyses are presented in Appendix D. Overall, most IRRs were close to 1. Subgroup analyses suggested some variability between intervention formats, with larger IRRs for partnership status and lower IRRs for self-efficacy in the blended counseling trial compared with the self-guided trial. Confidence intervals were generally wider in the blended counseling subsample, reflecting the smaller sample size.

### 3.3 Post-hoc power analyses

For multiple linear regression, assuming a medium effect size (*f*^2^ = 0.15), statistical power for the omnibus F-test was approximately 0.91 (*N* = 154, α = 0.05). In contrast, assuming a small effect (*f*^2^ = 0.02), power was substantially lower at approximately 0.15. For negative binomial regression, simulation-based analyses using 1000 simulations yielded a power of 0.87 for medium effects (*N* = 154, *α* = 0.05, *θ* = 1) and a power of 0.25 for small effects under the same assumptions.

### 3.4 Latent profile analysis

An exploratory latent profile analysis was conducted to identify empirically derived adherence patterns based on length, breadth, depth, and interaction. All indicators were log(x + 1)-transformed and z-standardized prior to analysis. Models with 1 to 4 latent classes were estimated with 200 random starts per solution. The 5-class model did not converge. The 4-class solution converged numerically but was rejected due to a near-empty smallest class (3.2%, n ≈ 5), indicating empirical underidentification given the sample size (n = 154).

Model selection was based on BIC and AIC as fit criteria, with minimum class size as an additional constraint. Both BIC and AIC decreased substantially from k = 1 to k = 3 (BIC: 1784.41 vs. 1290.00, ΔBIC = 494.41; AIC: 1760.12 vs. 1192.81, ΔAIC = 567.31) and then increased at k = 4 (BIC: 1350.09, ΔBIC = +60.09; AIC: 1207.36, ΔAIC = +14.55), yielding a clear minimum at k = 3 for both indices. The 3-class solution was therefore selected as the most parsimonious and well-supported solution, with an acceptable minimum class size of 15.6%. Entropy values exceeded 0.80 for both the 2- and 3-class solutions (0.940 and 0.952, respectively), indicating excellent separation between classes. Average posterior probabilities confirmed excellent classification accuracy across all three classes: Class 1 = 0.983, Class 2 = 0.977, Class 3 = 0.964.

As a sensitivity check, an alternative scaling approach was tested in which length was log-transformed and breadth, depth, and interaction were scaled to a 1–13 range to match the metric of the depth indicator (number of weeks following usage recommendations). Models with 1 to 5 classes were estimated. While AIC decreased continuously from 1-class (AIC: 2672.52) to 5-class (AIC: 2130.04), BIC, which penalizes model complexity more strongly, was lowest for the 3-class model (BIC: 2235.44). BIC was prioritized over AIC given its stronger penalty for model complexity, which is particularly appropriate at small sample sizes. The three-class solution demonstrated good classification certainty (entropy: 0.855), indicating a high degree of certainty in class assignments and well-separated latent classes. Average posterior probabilities of correct classification were lowest for Class 2 (0.921), followed by Class 1 (0.935) and Class 3 (0.983). Class sizes were 87 (56.5%) for Class 2, 42 (27.3%) for Class 1, and 25 (16.2%) for Class 3. These results converged with the primary analysis, supporting the robustness of the three-class solution.

Class 1, characterized by uniformly low values across all four indicators, was labeled *low adherence* (*n* = 37, 24.0%); Class 2, showing moderate values, *moderate adherence* (*n* = 93, 60.4%); and Class 3, showing the highest values across all indicators, *high adherence* (*n* = 24, 15.6%). Table 3 shows descriptive adherence statistics for each class, and class-specific adherence distributions are illustrated in Appendix E.

**Table 3:** Descriptive statistics of adherence indicators across latent profiles characterized by different levels of adherence.

	Low Adherence		Moderate Adherence		High Adherence	
Variable	*M*	*SD*	*M*	*SD*	*M*	*SD*
*N* (%)	37 (24.0%)		93 (60.4%)		24 (15.6%)	
Length	4.12	4.49	52.15	34.33	315.10	161.77
Breadth	1.08	1.36	17.65	17.39	71.00	18.04
Depth	0.11	0.31	1.80	1.91	6.12	3.29
Interaction	23.41	36.36	1021.44	1136.56	7839.50	5592.96

*Note*. *M* = mean; *SD* = standard deviation. Length was measured by the total time spent using the I-PREGNO app (in minutes), breadth by the number of completed sessions, depth by the number of weeks participants met the usage recommendation (at least three logins per week), interaction was measured as the number of characters entered in open text boxes.

Finally, a series of univariate multinomial logistic regression analyses were conducted to examine whether baseline psychosocial and demographic characteristics predicted latent class membership, with low adherers as the reference category. None of the predictors examined (depressive symptoms, parenting stress, difficulties in emotion regulation, self-efficacy, and partnership status) were significantly associated with class membership (see Table 4). Effect sizes were negligible, with McFadden's *R*^2^ ranging from 0.005 to 0.011, indicating that baseline characteristics accounted for only a minimal proportion of the variance in app usage class membership.

**Table 4:** Multinomial Logistic Regression Analyses Results.

	Low vs. Moderate Adherence				Low vs. High Adherence			
Predictors	*B*	*SE*	*p*	*OR [95% CI]*	*B*	*SE*	*p*	*OR [95% CI]*
Depressive symptoms	0.007	0.036	0.845	1.01 [0.94, 1.08]	0.062	0.046	0.179	1.06 [0.97, 1.17]
Parenting stress	−0.002	0.011	0.867	1.00 [0.98, 1.02]	0.018	0.015	0.234	1.02 [0.99, 1.05]
Difficulties in Emotion Regulation	0.005	0.017	0.790	1.00 [0.97, 1.04]	0.024	0.022	0.275	1.02 [0.98, 1.07]
Self-efficacy	−0.118	0.396	0.766	0.89 [0.41, 1.93]	−0.832	0.524	0.113	0.44 [0.16, 1.22]
Partnershipᵃ	0.992	0.736	0.178	2.70 [0.64, 11.41]	1.025	1.151	0.373	2.79 [0.29, 26.59]

*Note.* Reference category = Low Adherence. Depressive symptoms: EPDS; Parenting stress: PSI-SF; Difficulties in Emotion Regulation: DERS-SF; Self-efficacy: SWE.

ᵃ 0 = no partner, 1 = has partner. *B* = regression coefficient (log odds); *SE* = standard error; OR = odds ratio. *p* < .05 considered significant.

## 4 Discussion

This study examined whether psychological characteristics predict multidimensional adherence to the I-PREGNO app among postpartum mothers and whether distinct adherence patterns could be identified. Although initial regression analyses suggested differential associations across adherence dimensions, none of these effects remained statistically significant after controlling for multiple testing using FDR adjustment. In addition, exploratory latent profile analysis identified three adherence patterns (low, moderate, and high) indicating meaningful heterogeneity in how mothers used the app. However, psychological characteristics did not significantly predict adherence class membership, suggesting that psychological factors may play a limited role in explaining overall adherence patterns.

### 4.1 Regression analyses

Overall, model performance was weak across all four adherence dimensions, with little to no improvement over null models. Contrary to hypotheses, depressive symptoms, parenting stress, emotion regulation difficulties, self-efficacy, and partnership status did not significantly predict any adherence dimension after correction for multiple testing. Parenting stress and emotion regulation difficulties showed no meaningful associations even before correction, despite theoretical expectations that these factors would constrain engagement through reduced motivational capacity, limited self-regulatory resources, and competing situational demands (Jakob et al., 2022; Makama et al., 2021).

For the remaining predictors, initial associations were dimension-specific. Higher depressive symptoms were initially associated with greater interaction, consistent with some mHealth findings (Mikolasek et al., 2018). However, this association was further attenuated in sensitivity analyses, suggesting the models may be partly driven by a small number of extreme observations. Lower self-efficacy was initially associated with more weeks following the recommendation of using the app at least three times a week (depth), contrary to theoretical expectations and previous findings suggesting positive associations with adherence (Jakob et al., 2022). After correction for multiple testing, this pattern was no longer evident and is more consistent with previously reported negligible associations (Beatty and Binnion, 2016). Partnership status was initially associated with longer app use, in line with literature highlighting the facilitating role of social support in postpartum populations (Beatty and Binnion, 2016; Liu et al., 2022; Reinwand et al., 2015). However, this association did not survive FDR correction, which may partly reflect restricted variance, as more than 90% of participants reported being in a partnership.

These findings are broadly consistent with meta-analytic and systematic evidence indicating that user characteristics typically explain only modest proportions of variance in digital intervention adherence and show mixed directional associations across studies (Kelders et al., 2013; Figueiredo et al., 2025). The results suggest that baseline psychological characteristics carry limited explanatory value for adherence variability in I-PREGNO. Rather than reflecting a true absence of association, the weak and inconsistent findings may indicate that any effects are small in magnitude and therefore difficult to detect reliably in the present sample. They may also reflect the limitations of static baseline measurement in a context where adherence is likely shaped by rapidly shifting postpartum demands and dynamic in-app experiences that are not captured by trait-level assessments.

### 4.2 Latent profile analysis

LPA identified three adherence patterns (low, moderate, and high) consistent across dimensions, indicating interdependence among adherence measures. Most participants showed moderate adherence, with high adherence being rare, consistent with prior findings (Gilmore et al., 2017). Some mothers demonstrated low adherence, frequently failing to follow usage recommendations. This suggests that while mHealth interventions like I-PREGNO may initially attract interest, many users fail to fully engage with them. Furthermore, none of the examined factors including depressive symptoms, parenting stress, difficulties in emotion regulation, self-efficacy, and partnership status showed a significant link to adherence patterns. These findings imply that the psychological variables measured may not be key determinants of overall adherence in this sample, and that other unmeasured factors, such as external barriers or the intervention's design, could play a more significant role in influencing user adherence in postpartum mothers.

### 4.3 Limitations

Post-hoc power analyses indicated adequate power to detect medium-sized effects but limited power for small effects. Given that adherence predictors explained only small proportions of variance, true associations are likely to be small and therefore difficult to detect reliably in the present sample.

Several methodological factors may have further influenced the detection and interpretation of effects. *P*-values were adjusted using FDR correction to reduce the risk of false positives; however, this may have increased the likelihood of false negatives and obscured small but potentially meaningful associations.

The study design also introduces constraints related to model specification and generalizability. The pooling of two distinct RCT contexts (blended counseling and self-guided use) implies that estimates reflect average associations across heterogeneous delivery formats. Although intervention type was included as a covariate, the effects may still be shaped by context-dependent mechanisms of adherence. The current sample size did not allow for stable estimation of interaction effects, leaving potential moderation by intervention context an open question. Subgroup analyses were exploratory and limited by small sample size, particularly in the blended counseling group, resulting in imprecise estimates. Across predictors and adherence dimensions, no consistent pattern of differences between contexts emerged.

Analytically, the selection of five psychological factors involved a trade-off between theoretical coverage and statistical feasibility. This approach allowed us to capture key psychological influences without overcomplicating the model. However, including additional predictors (e.g., technological, temporal, or social factors) might have increased explanatory power, while also raising risks of overfitting, multicollinearity, and reduced model stability.

Although objective usage data improve comparability and reduce bias compared to self-report (Yang et al., 2022), future studies would benefit from integrating qualitative and quantitative approaches to obtain a more comprehensive understanding of adherence processes (Kernebeck et al., 2021).

Finally, adherence in postpartum populations may be particularly dynamic, shaped by rapidly changing daily circumstances (e.g., infant sleep patterns, caregiving demands, return to work) that are difficult to capture with baseline assessments. Consequently, static psychological measures may be less sensitive to engagement than time-varying contextual factors.

### 4.4 Implications

The present findings carry implications for both research and practice in perinatal mHealth. Given the generally small and non-robust associations observed, psychological characteristics alone may be insufficient to explain variability in adherence. Future work should therefore consider broader multilevel frameworks that integrate contextual, social, and intervention-related determinants. In the postpartum context, factors such as time constraints, caregiving demands, perceived relevance of app components, and usability may represent more proximal drivers of engagement than stable individual traits. Examining how these factors relate to different adherence dimensions may help clarify heterogeneous usage patterns, including early disengagement or selective use.

At the conceptual level, results support treating adherence as a multidimensional construct. The observed interrelations among adherence dimensions and the identification of low-, moderate-, and high-adherence patterns suggest meaningful heterogeneity in how mothers engaged with the app. Future research should therefore further differentiate between adherence dimensions and examine whether specific predictors are differentially associated with distinct adherence dimensions to the intervention (e.g., length, breadth, depth, and interaction). Beyond conceptual differentiation, however, the question of what drives variability across these dimensions remains largely unresolved.

The identification of distinct adherence patterns further suggests that future studies should complement variable-centered approaches with person-centered analyses. Larger samples would enable more stable profile solutions and allow systematic examination of predictors of class membership, including potential moderation by intervention context or demographic characteristics. Ultimately, a better understanding of what distinguishes different adherence subgroups could inform how perinatal mHealth interventions are tailored, e.g., by identifying which users are at risk of early disengagement and targeting support accordingly.

Finally, adequately powered studies are needed given that true associations are likely small, and complementary qualitative approaches may further elucidate which barriers are most salient in real-world postpartum mHealth adherence. Taken together, the results suggest that psychological factors derived from motivational, self-regulatory, and resource-related frameworks (Bina, 2020; Schwarzer, 2008; West and Michie, 2020; Yeager and Benight, 2018) showed weak and inconsistent associations with adherence and may be insufficient when operationalized as static baseline measures in a rapidly changing postpartum context. Adherence in perinatal mHealth may therefore be better understood through dynamic, multilevel models that capture both individual characteristics and the evolving circumstances in which app use occurs.

## 5 Conclusion

This study examined whether psychological characteristics predict multidimensional adherence to the I-PREGNO mHealth app among postpartum mothers and whether distinct adherence patterns could be identified. Overall, no psychological baseline variables significantly predicted adherence after correction for multiple testing, and no robust associations were observed across adherence dimensions. Exploratory latent profile analysis identified three adherence patterns (low, moderate, and high), indicating heterogeneity in app use within the sample. However, these patterns were not explained by the psychological factors assessed.

Taken together, the findings suggest that baseline psychological characteristics have limited utility for explaining variability in mHealth adherence among postpartum mothers. Future research should therefore adopt a broader perspective by examining social, structural, and technological determinants of engagement, such as time constraints, resource availability, app usability, and intervention design features. In addition, further work may benefit from conceptualizing adherence as a multidimensional construct to better capture nuanced usage patterns and their determinants in larger and more diverse samples.

## Abbreviations

App Application

BMI Body Mass Index

cRCT Cluster randomized controlled trial

DERS-SF Difficulties in Emotion Regulation Scale

EPDS Edinburgh-Postnatal-Depression-Scale

GSE General Self-Efficacy Scale

LPA Latent Profile Analysis

mHealth Mobile health

PSI-SF Parenting Stress Index

RCT Randomized controlled trial

## Author contributions

LV, TD, JL, UL, AO, CS, JW, CH designed the study. NS, JH, LV, AO, CS, CH collected the data. NS and JH performed the data analysis, data interpretation, and generation of figures and tables. NS, JH and CH drafted the manuscript. LV, TD, JL, UL, CS, JW contributed to the final version of the manuscript. All authors reviewed and approved the final manuscript.

## Trial registrations

Trials were registered prospectively at the German Clinical Trials Register (blended counselling in July 2022: DRKS00029673; self-guided in January 2023: DRKS00031067)

## Declaration of Generative AI and AI-assisted technologies in the writing process

During the preparation of this work the authors used ChatGPT/ Claude/ DeepL to improve the readability and language of the manuscript. After using these tools, the authors reviewed and edited the content as needed and take full responsibility for the content of the published article.

## Funding

This project has received funding from the European Union's Horizon 2020 research and innovation programme under the ERA-NET Cofund action N° 727565 and is part of the “European Joint Programming Initiative A Healthy Diet for a Healthy Life" (JPI HDHL). The study is supported by the 10.13039/501100002347German Federal Ministry of Education and Research under transaction number 01EA2103A, by the 10.13039/501100004955Austrian Research Promotion Agency (FFG) under grant number 883212, and by the 10.13039/501100003130Research Foundation Flanders (FWO) under grant number G0H8520N. The authors acknowledge support by the Open Access Publication Fund of the University of Bamberg. L.V. is supported by the German Center for Mental Health (DZPG) funded by the 10.13039/501100002347Federal Ministry of Education and Research (Bundesministerium für Bildung und Forschung [BMBF]) and the ministry of Bavaria (01EE2303A). C.S. is supported by the DZPG funded by the 10.13039/501100002347BMBF (01EE2301C).

## Declaration of competing interest

The authors declare that they have no known competing financial interests or personal relationships that could have appeared to influence the work reported in this paper.

## Figures and Tables

**Table:** 

Module	Session
Tutorial	1. Objectives and content of the app 2. Usage of the app
Introduction	1. Psychoeducation”self-care as a parent-(to-be)” 2. Importance of self-care (i.e., mental wellbeing, nutrition, exercise behavior) for parents 3. Changes during the pregnancy and postpartum period 4. Goal setting
Self-esteem	1. Psychoeducation “self-esteem” 2. Identifying personal resources 3. Strengthening personal resources 4. Developing new personal resources 5. Developing self-esteem-enhancing cognitions 6. Implementing self-esteem-enhancing cognitions 7. Implementing a daily self-esteem journal 8. Psychoeducation “development of children's self-esteem” 9. Changes of the body 10. Dealing with difficult situations in a self-assured manner 11. Introduction “body image” 12. Cognitive restructuring 13. Identifying cognitive distortions 14. Reducing avoidance behavior 15. Developing self-reward techniques 16. Conclusion “personal learning in this module”
Stress management	1. Psycho-education “stress” 2. Identifying individual stressors 3. Identifying individual stress reactions 4. Introduction of stress management skills 5. Problem solving exercise 6. Time management exercise 7. Breath relaxation exercise 8. Meditation exercise “walking” 9. Mindfulness exercise “sense of touch” 10. Mindfulness exercise “tasting” 11. Mindfulness exercise “perception walk” 12. Meditation exercise “sitting” 13. Mindfulness exercise “sense of smell” 14. Cognitive exercise “packing parcels” 15. Radical acceptance 16. Sleep as a special stressor 17. Taking responsibility for tasks 18. Handing over responsibility for tasks 19. Conclusion „personal learning from this module“
Emotion regulation	1. Psychoeducation “functions of emotions” 2. Psychoeducation “development of emotions” 3. Relation between emotions and cognitions 4. Recognizing and defusing cognitive distortions 5. Introduction of emotion regulation skills 6. Emotion surfing exercise 7. Psychoeducation “emotions and hormones” 8. Dealing with depressive moods
	9. Emotion regulation in infants 10. Relation between physical activity and emotions 11. Mindfulness exercise “walking” 12. Mindfulness exercise “Five senses” 13. Focused attention exercise 14. Developing helpful emotional beliefs 15. Promoting self-compassion 16. Behaving contrary to the emotion 17. Implementing distraction 18. Identifying positive activities 19. Conclusion “personal learning from this module”
Self-efficacy	1. Psychoeducation “self-efficacy” 2. Identifying sources of self-efficacy 3. Identifying daily personal achievements 4. Coping with success and failure 5. Personal attribution of success and failure 6. Identifying role models 7. Discovering personal resources 8. Improving self-efficacy of children 9. Conclusion “personal learning from this module”
Social competences	1. Psychoeducation “social relations”, identifying social resources 2. Psychoeducation “change in social relationships within and after the pregnancy” 3. Observing without judging 4. Recognizing and expressing feelings 5. Recognizing and communicating personal needs 6. Asking other people for something 7. Conclusion “personal learning from this module”
Mindfulness	1. Psychoeducation “mindfulness” 2. Identifying relations between mindfulness and mental health 3. Meditation exercises for the pregnancy and postpartum period 4. Introduction of further mindfulness exercises 5. Conclusion “personal learning from this module”
Nutrition	1. Psychoeducation “Nutrition and weight development” 2. Finding out why water is valuable 3. Discovering how a diverse plate looks like 4. Discovering vegetable food 5. Dealing with sweets and fast-food 6. Finding the good portion of meat 7. Breastfeeding
Physical activity	1. Psychoeducation “physical activity during the pregnancy/ postpartum period” 2. Monitoring and altering sitting behavior 3. Psychoeducation “weight training during the pregnancy/ postpartum period” 4. Strength exercises: training notes 5. Strength exercises “upper body” 6. strength exercises “full body” 7. strength exercises “lower body” 8. strength exercises “pelvic floor”
Self-monitoring	/
Conclusion	1. Summarizing and reflecting on the app content 2. Review of goal achievement 3. Sticking to the personal goals 4. Dealing with setbacks

*Note.* Adapted from “User-centered development process of an evidence-based mHealth intervention for psychosocially burdened families during the transition to parenthood,” by L. Vogel, C. Henning, J. Wolstein, V. Versele, M. N. M. Van Poppel, K. Steppan, T. Schlossbach, A. Opitz, U. Lux, J. Löchner, T. Färber, T. Deliens, E. Boehlke and C. Seiferth, 2024, *Mental Health Science*, *2*(2), Article e58 (doi:https://doi.org/10.1002/mhs2.58). Copyright 2024 by the Authors. Mental Health Science published by Wiley Periodicals LLC.

**Table:** 

Package	Purpose of use	Source
haven (V. 2.5.4)	importing data	Wickham et al. (2023)
readxl (V. 1.4.3)	importing data	Wickham and Bryan (2023))
tidyverse (V. 2.0.0)	data preparation	Wickham et al. (2019)
psych (V. 2.4.3)	descriptive statistics	Revelle (2024)
arsenal (V. 3.6.3)	descriptive statistics	Heinzen et al. (2021)
correlation (V. 0.8.4)	correlation analysis	Makowski et al. (2020)
olsrr (V. 0.6.0)	checking regression assumptions	Hebbali (2024)
lmtest (V. 0.9–40)	checking regression assumptions	Zeileis and Hothorn (2002)
car (V. 3.1–2)	checking regression assumptions	Fox and Weisberg (2019)
robust (V. 0.7–4)	fitting robust multiple linear regression	Wang et al. (2024)
MASS (V. 7.3–60.0.1)	fitting negative binomial regression models	Venables and Ripley (2002)
rcompanion (V. 2.4.35)	calculating pseudo R squared for negative binomial regression and effect sizes for difference analyses	Mangiafico (2024)
performance (V. 0.11.0)	checking assumptions for negative binomial regression models, checking zero-inflation	Lüdecke et al. (2021a)
see (V. 0.8.4)	visualizing assumptions for negative binomial regression models	Lüdecke et al. (2021b)
DHARMa (V. 0.4.6)	checking assumptions for negative binomial regression models (residual diagnostics)	Hartig (2022)
tidyLPA (V. 1.1.0)	latent profile analysis	Rosenberg et al. (2018)
depmixS4 (V. 1.5–0)	latent profile analysis	Visser and Speekenbrink (2010)
nnet (V. 7.3–20)	multinomial logistic regression analysis	Venables and Ripley (2002)

*Note. V.* = Version.

**Table C1:** Results of the Robust Regression for the log-transformed Adherence Dimension Length.

Predictors	Length Model 1						Length Model 2					
	*B*	*SE B*	95% CI for *B*	β	*t*	*p*	*B*	*SE B*	95% CI for *B*	β	*t*	*p*
Constant	2.74	2.23	[*−*1.62, 7.10]		1.23	0.220	2.38	2.11	[−1.76, 6.53]		1.13	0.262
Control variables												
Age	0.03	0.06	[−0.80, 0.15]	0.11	0.58	0.563	0.01	0.03	[−0.06, 0.08]	0.03	0.24	0.815
Education (reference: high education)												
Medium education a	−0.26	0.71	[−1.65, 1.12]	–0.08	−0.37	0.712	−0.43	0.42	[−1.24, 0.39]	–0.12	−1.03	0.304
Low education a	−1.01	1.22	[−3.39, 1.37]	–0.20	−0.83	0.407	−0.51	0.72	[−1.93, 0.90]	–0.10	−0.71	0.477
BMI	−0.01	0.05	[−0.10, 0.09]	–0.02	−0.13	0.895	−0.02	0.03	[−0.08, 0.04]	–0.07	−0.57	0.569
Age of the child	−0.01	0.08	[−0.17, 0.16]	–0.01	−0.06	0.950	−0.01	0.05	[−0.11, 0.09]	–0.02	−0.18	0.858
Type of intervention b	0.75	0.79	[−0.81, 2.30]	0.20	0.94	0.348	0.76	0.50	[−0.22, 1.74]	0.20	1.52	0.130
Psychological variables												
Depressive symptoms							0.05	0.05	[−0.05, 0.14]	0.16	0.96	0.338
Parenting stress							0.01	0.01	[−0.02, 0.03]	0.07	0.47	0.640
Difficulties in emotion regulation							−0.02	0.02	[−0.06, 0.02]	–0.12	−0.78	0.435
Self-efficacy							−0.20	0.35	[−0.89, 0.49]	–0.06	−0.57	0.570
Partnership a							1.90⁎	0.80	[0.32, 3.47]	0.28⁎	2.36	0.020
*R*^*2*^	0.035						0.092					
Δ*R*^*2*^							0.057					

*Note*. *N* = 154. CI = confidence interval; incl. = inclusion.

a 0 = no, 1 = yes.

b 0 = application access without blended counseling, 1 = application access including blended counseling.

⁎ *p* < .05.

**Table C2:** Results of the Negative Binomial Regression for the Adherence Dimension Breadth.

Predictors	Breadth Model 1						Breadth Model 2					
	*B*	*SE B*	IRR	95% CI for IRR	*z*	*p*	*B*	*SE B*	IRR	95% CI for IRR	*z*	*p*
Constant	2.63⁎⁎	0.92			2.86	0.004	1.14	1.44			0.79	0.427
Control variables												
Age	0.02	0.02	1.02	[0.97, 1.07]	0.73	0.468	0.02	0.02	1.02	[0.97, 1.07]	0.69	0.490
Education (reference: high education)												
Medium education a	−0.02	0.29	0.98	[0.54, 1.81]	−0.07	0.942	0.01	0.28	1.01	[0.55, 1.88]	0.04	0.968
Low education a	−0.25	0.49	0.78	[0.29, 2.14]	−0.52	0.605	−0.20	0.50	0.82	[0.29, 2.44]	−0.39	0.695
BMI	−0.00	0.02	1.00	[0.96, 1.04]	−0.23	0.816	−0.00	0.02	1.00	[0.96, 1.04]	−0.10	0.924
Age of the child	−0.01	0.03	0.99	[0.93, 1.06]	−0.27	0.785	−0.01	0.03	0.99	[0.92, 1.06]	−0.33	0.742
Type of intervention b	0.35	0.32	1.42	[0.74, 2.86]	1.09	0.274	0.21	0.35	1.23	[0.59, 2.68]	0.61	0.545
Psychological variables												
Depressive symptoms							0.02	0.03	1.02	[0.95, 1.09]	0.52	0.607
Parenting stress							0.01	0.01	1.01	[1.00, 1.03]	1.63	0.104
Difficulties in emotion regulation							−0.01	0.01	0.99	[0.96, 1.02]	−0.68	0.496
Self-efficacy							0.13	0.24	1.14	[0.68, 1.91]	0.54	0.587
Partnership a							0.29	0.53	1.34	[0.39, 3.85]	0.55	0.580
Dispersion (θ)	0.59						0.60					
2 x log-likelihood	−1239.36						−1235.24					
Mc Fadden R^2^/Efron R^2^	0.001/0.007						0.005/0.019					

*Note*. *N* = 154. IRR = incidence rate ratio; CI = confidence interval; incl. = inclusion.

a 0 = no, 1 = yes.

b 0 = application access without blended counseling, 1 = application access including blended counseling.

⁎⁎ *p* < .01.

**Table C3:** Results of the Negative Binomial Regression Models for the Adherence Dimension Depth.

Predictors	Depth Model 1						Depth Model 2					
	*B*	*SE B*	IRR	95% CI for IRR	*z*	*p*	*B*	*SE B*	IRR	95% CI for IRR	*z*	*p*
Constant	−0.10	0.85			−0.12	0.906	0.12	1.32			0.09	0.928
Control variables												
Age	0.03	0.02	1.04	[0.99, 1.08]	1.59	0.111	0.05⁎	0.02	1.05⁎	[1.01, 1.10]	2.33	0.020
Education (reference: high education)												
Medium education a	−0.14	0.27	0.87	[0.52, 1.48]	−0.51	0.612	−0.04	0.27	0.96	[0.57, 1.61]	−0.17	0.866
Low education a	−0.00	0.45	1.00	[0.44, 2.31]	−0.00	0.998	0.16	0.46	1.18	[0.50, 2.84]	0.36	0.723
BMI	−0.01	0.02	0.99	[0.96, 1.03]	−0.49	0.621	−0.02	0.02	0.98	[0.94, 1.02]	−1.01	0.311
Age of the child	−0.03	0.03	0.97	[0.91, 1.03]	−0.99	0.321	−0.03	0.03	0.97	[0.91, 1.03]	−1.10	0.270
Type of intervention b	0.41	0.30	1.50	[0.85, 2.69]	1.36	0.173	0.45	0.32	1.57	[0.84, 2.97]	1.43	0.153
Psychological variables												
Depressive symptoms							−0.02	0.03	0.98	[0.92, 1.04]	−0.72	0.473
Parenting stress							−0.00	0.01	1.00	[0.98, 1.01]	−0.15	0.881
Difficulties in emotion regulation							0.02	0.01	1.02	[0.99, 1.04]	1.23	0.220
Self-efficacy							−0.45⁎	0.22	0.64⁎	[0.40, 1.02]	−2.01	0.044
Partnership a							0.46	0.51	1.58	[0.58, 4.22]	0.90	0.370
Dispersion (θ)	0.99						1.10					
2 x log-likelihood	-590.23						−581.12					
Mc Fadden R^2^/Efron R^2^	0.010/0.029						0.025/0.062					

*Note*. *N* = 154. IRR = incidence rate ratio; CI = confidence interval; incl. = inclusion.

a 0 = no, 1 = yes.

b 0 = application access without blended counseling, 1 = application access including blended counseling.

⁎ *p* < .05.

**Table C4:** Results of the Negative Binomial Regression for the Adherence Dimension Interaction.

Predictors	Interaction Model 1														Interaction Model 2													
	*B*		*SE B*	IRR		95% CI for IRR					*z*		*p*		*B*		*SE B*	IRR		95% CI for IRR					*z*		*p*	
Constant	7.49	⁎⁎⁎	1.22								6	0.14	<	0.001	8.57	⁎⁎⁎	1.90								4	0.51	<	0.001
Control variables																												
Age	0.04		0.03		1.04	[	0.97	,	1	0.11]	1	0.28		0.202	0.02		0.03		1.02	[	0.95	,	1	0.10]	0	0.73		0.469
Education (reference: high education)																												
Medium education a	0.02		0.38		1.02	[	0.46	,	2	0.28]	0	0.05		0.958	−0.01		0.38		0.99	[	0.45	,	2	0.23]	−0	0.01		0.989
Low education a	−1.12		0.65		0.33	[	0.10	,	1	0.14]	−1	0.73		0.084	−1.49	⁎	0.66		0.23⁎	[	0.07	,	0	0.86]	−2	0.25		0.025
BMI	−0.04		0.03		0.96	[	0.91	,	1	0.02]	−1	0.65		0.100	−0.06	⁎	0.03		0.94⁎	[	0.89	,	1	0.01]	−2	0.06		0.039
Age of the child	−0.07		0.04		0.93	[	0.85	,	1	0.02]	−1	0.64		0.102	−0.07		0.04		0.93	[	0.85	,	1	0.02]	−1	0.68		0.094
Type of intervention b	0.78		0.42		2.18	[	1.01	,	5	0.06]	1	0.84		0.067	0.38		0.46		1.47	[	0.59	,	3	0.84]	0	0.85		0.398
Psychological variables																												
Depressive symptoms															0.10	⁎	0.04		1.10⁎	[	1.00	,	1	0.21]	2	0.16		0.031
Parenting stress															−0.01		0.01		0.99	[	0.97	,	1	0.02]	−0	0.72		0.472
Difficulties in emotion regulation															0.00		0.02		1.00	[	0.96	,	1	0.05]	0	0.11		0.915
Self-efficacy															−0.01		0.32		0.99	[	0.49	,	2	0.04]	−0	0.04		0.972
Partnership a															−0.27		0.69		0.76	[	0.16	,	2	0.61]	−0	0.40		0.693
Dispersion (θ)	0.33														0.34													
2 x log-likelihood	−2409.16														−2402.68													
Mc Fadden R^2^ / Efron R^2^	0.004/0.029														0.007/0.081													

*Note*. *N* = 154. IRR = incidence rate ratio; CI = confidence interval; incl. = inclusion.

a 0 = no, 1 = yes.

b 0 = application access without blended counseling, 1 = application access including blended counseling.

⁎ *p* < .05.

⁎⁎⁎ *p* < .001.

**Table D1:** Standardized Regression Coefficients for Baseline Predictors of Length in Pooled and Subsample Analyses.

Predictors	Pooled adjusted *β* [95% CI] (*N* = 154)	Pooled unadjusted *β* [95% CI] (*N* = 154)	Blended Counseling *β* [95% CI] (*n* = 37)	Self-guided *β* [95% CI] (*n* = 117)
Depressive symptoms	0.16 [−0.17, 0.49]	0.14 [−0.08, 0.37]	0.14 [−0.26, 0.54]	0.07 [−0.27, 0.41]
Parenting stress	0.07 [−0.21, 0.34]	0.12 [−0.05, 0.30]	0.06 [−0.32, 0.43]	0.12 [−0.11, 0.36]
Difficulties in emotion regulation	−0.12 [−0.42, 0.18]	0.06 [−0.16, 0.28]	0.03 [−0.39, 0.46]	0.04 [−0.26, 0.35]
Self-efficacy	−0.06 [−0.28, 0.15]	−0.11 [−0.32, 0.10]	−0.14 [−0.53, 0.25]	−0.07 [−0.37, 0.23]
Partnership a	0.28 [0.05, 0.50]	0.26 [−0.00, 0.51]	0.13 [−0.21, 0.47]	−0.43 [−3.02, 2.16]

*Note.* Pooled adjusted model includes all covariates and psychological predictors simultaneously (robust linear regression). Unadjusted estimates are univariate standardized betas. Within-trial estimates are based on univariate robust linear models. *n* = sample size per subsample.

a 0 = no, 1 = yes.

**Table D2:** Negative Binomial Regression Models of Adherence (Breadth, Depth, and Interaction): Pooled and Subsample Analyses.

Predictors	Pooled adjusted IRR [95% CI] (*N* = 154)	Pooled unadjusted IRR [95% CI] (*N* = 154)	Blended Counseling IRR [95% CI] (*n* = 37)	Self-guided IRR [95% CI] (*n* = 117)
Breadth				
Depressive symptoms	1.02 [0.95, 1.09]	1.03 [0.99, 1.07]	1.06 [0.99, 1.14]	1.01 [0.96, 1.07]
Parenting stress	1.01 [1.00, 1.03]	1.01 [1.00, 1.02]	1.01 [0.99, 1.03]	1.01 [0.99, 1.03]
Difficulties in emotion regulation	0.99 [0.96, 1.02]	1.01 [0.99, 1.03]	1.02 [0.98, 1.05]	1.00 [0.98, 1.02]
Self-efficacy	1.14 [0.68, 1.91]	0.89 [0.60, 1.35]	0.80 [0.44, 1.52]	1.00 [0.57, 1.75]
Partnership a	1.34 [0.39, 3.85]	1.18 [0.42, 2.63]	1.68 [0.49, 4.54]	0.75 [0.06, 3.19]
Depth				
Depressive symptoms	0.98 [0.92, 1.04]	1.02 [0.99, 1.06]	1.06 [1.00, 1.13]	0.99 [0.95, 1.05]
Parenting stress	1.00 [0.98, 1.01]	1.01 [1.00, 1.02]	1.00 [0.99, 1.02]	1.01 [0.99, 1.02]
Difficulties in emotion regulation	1.02 [0.99, 1.04]	1.02 [1.00, 1.03]	1.02 [0.99, 1.05]	1.01 [0.99, 1.04]
Self-efficacy	0.64 [0.40, 1.02]	0.62 [0.42, 0.93]	0.51 [0.27, 0.93]	0.74 [0.43, 1.25]
Partnership a	1.58 [0.58, 4.22]	1.46 [0.58, 3.50]	3.87 [1.21, 13.62]	0.48 [0.07, 2.00]
Interaction				
Depressive symptoms	1.10 [1.00, 1.21]	1.06 [1.01, 1.12]	1.13 [0.99, 1.30]	1.05 [0.99, 1.13]
Parenting stress	0.99 [0.97, 1.02]	1.01 [0.99, 1.03]	1.01 [0.98, 1.04]	1.01 [0.99, 1.04]
Difficulties in emotion regulation	1.00 [0.96, 1.05]	1.02 [0.99, 1.05]	1.01 [0.95, 1.09]	1.02 [0.99, 1.05]
Self-efficacy	0.99 [0.49, 2.04]	0.77 [0.39, 1.52]	0.61 [0.20, 1.87]	0.94 [0.39, 2.31]
Partnership a	0.76 [0.16, 2.61]	1.22 [0.28, 3.43]	10.56 [2.00, 36.70]	0.28 [0.00, 1.69]

*Note.* Pooled adjusted model includes all covariates and psychological predictors simultaneously (negative binomial regression). Pooled unadjusted estimates are univariate incidence rate ratios. Within-trial estimates are based on univariate negative binomial models within each subsample. Subsample models are unadjusted due to limited sample size. *n* = sample size per subsample.

a 0 = no, 1 = yes.

**Figure:**
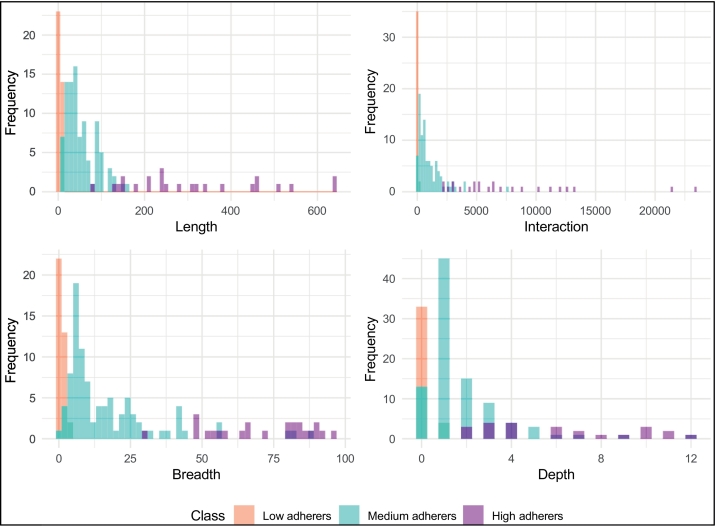


## Data Availability

I-PREGNO data will be published on the OSF platform as part of the I-PREGNO project by the end of 2026. If there is a legitimate interest in the data in advance, the data can be requested from the corresponding author.
